# Healthcare Workers From Two Sites in China in 2018–2019 Unlikely to Receive and Recommend Influenza Vaccination: A Qualitative Study Following a Quantitative Analysis to Improve Future Interventions

**DOI:** 10.1111/irv.70157

**Published:** 2025-11-02

**Authors:** Jing Fan, Ran Zhang, Qianyue Zhang, Shu Cong, Ying Song, W. William Schluter, Suizan Zhou, Xuping Song, Wenjing Wang, Liwen Fang

**Affiliations:** ^1^ National Center for Chronic and Non‐Communicable Disease Control and Prevention Chinese Center for Disease Control and Prevention Beijing China; ^2^ Centers for Disease Control and Prevention Atlanta Georgia USA; ^3^ School of Mathematics and Statistics Wuhan University Wuhan China

**Keywords:** healthcare worker, influenza vaccination, recommendation

## Abstract

**Background:**

The proportion of healthcare workers (HCWs) who receive and recommend seasonal influenza vaccination to their patients remains low in China. This study aims to understand why HCWs infrequently use and recommend the influenza vaccine and how to improve utilization.

**Methods:**

A cross‐sectional questionnaire survey and a focus group interview were conducted among primary HCWs in Hubei Province in September 2018 and May 2019. We analyzed qualitative data using descriptive methods and a general inductive approach following a quantitative analysis. In addition, we used the Health Belief Model (HBM) framework to summarize predictors of HCW vaccination and recommendation.

**Results:**

Primary HCWs acquired basic knowledge about influenza infection and vaccination and were less likely to receive and recommend influenza vaccination. However, from the focus group, HCWs reported influenza was a mild disease and would not recommend vaccination for patients who looked healthy. HCWs raised concerns about adverse events, cost‐effectiveness, and contraindications to influenza vaccination. HCWs reported, “I would be more likely to recommend vaccination if my employer required that I do so.”

**Conclusions:**

Health education materials for HCWs could be improved by providing scientific evidence on the burden of influenza disease, the benefits of vaccination, and national and international policies on influenza vaccination. In addition, interventions that may improve influenza vaccination coverage include workplace requirements for influenza vaccination of HCWs and requirements for HCWs to recommend influenza vaccination to high‐risk groups in addition to providing no‐cost and on‐site vaccination.

## 1 Introduction

Healthcare workers (HCWs) are at increased risk of occupational exposure to and infection with influenza [1]. In addition, HCWs may themselves serve as potential carriers for healthcare transmission of influenza to vulnerable patients [2, 3]. HCW absenteeism due to influenza may burden healthcare systems and reduce availability of essential services during influenza outbreaks and pandemics. Influenza vaccination of HCWs is a key strategy to prevent HCW infection and absenteeism, healthcare transmission, and associated morbidity and mortality of vulnerable patients [4, 5, 6, 7]. In addition, vaccinated HCWs are more likely to recommend influenza vaccination to their patients and play a vital role in increasing influenza vaccine uptake among other high‐risk populations [8]. However, influenza vaccination coverage among HCWs in China is low. Many factors contribute to the low coverage, such as misconceptions among HCWs about the severity of influenza disease and the risks of vaccination. Also, there is a lack of accessible workplace vaccination programs because vaccine clinics are usually established in community health centers, and on‐site vaccination is not available for most HCWs working at general hospitals. In addition, HCWs need to pay out of pocket (about 10–15 USD) for the vaccine, and limited supply may contribute to the low uptake in some seasons.

The Health Belief Model (HBM) is the most frequently used theory to predict influenza vaccination uptake among HCWs [9]. Earlier studies have applied the HBM model to identify predictors of influenza vaccination. However, gaps exist between identifying predictors and designing effective interventions. Though influenza vaccine campaigns have been implemented annually in China, our previous program found that 13.6% (122/896) primary HCWs were vaccinated and 17.9% (160/896) of primary HCWs recommended influenza vaccination to their patients in Hubei during the influenza season 2017–2018 [10]. To dig into the reasons for HCWs' conceptions on influenza vaccination and recommendation and inform interventions, we conducted a follow‐up focus group discussion. In this paper, we explore an approach of using the HBM model and analyzing quantitative and qualitative data to identify specific predictors linked to interventions to change HCW behavior related to influenza vaccination and recommendation of vaccination for their patients.

## 2 Materials and Methods

This study collected quantitative and qualitative data from Fancheng District of Xiangyang City and Chibi County of Hubei Province in China. The procedure of quantitative data collection and characteristics of participants was described in a previous paper [10]. We selected primary healthcare clinics including community health centers/branches in the urban areas and township hospitals/village clinics in the rural areas that provide primary healthcare services in China. Primary HCWs are defined as doctors and nurses, including clinical doctors and nurses, as well as public health doctors and nurses. We enrolled all categories of primary HCWs because they all have the opportunity to recommend influenza vaccination to their patients during health service in China. Specifically, at the primary healthcare setting, clinical doctors include general practitioners and specialists (such as Chinese Traditional Medicine doctors), who provide clinical diagnosis and treatment as well as health education to patients, and clinical nurses aid clinical doctors during the course of clinical practice. In addition, a public health doctor or nurse is a professional category with defined public health responsibility for disease prevention, health promotion and education, epidemic control, and the implementation of public health policies in China. All primary HCWs of the selected clinics were recruited according to the name list of employees in September 2018. No exclusion criteria were defined. After obtaining informed consent, a standardized questionnaire was used to collect information on demographic characteristics, attitudes, and practices for influenza vaccination and recommendation. The three most frequently mentioned reasons for receiving vaccination or recommending influenza vaccinations for patients were collected. Data were analyzed using descriptive statistics with SAS Version 9.4.

We also conducted focus group interviews of HCWs using a semistructured questionnaire in May 2019. HCWs were classified into three categories based on their recommendations for influenza vaccination and vaccine acceptance of corresponding patients: (1) HCWs with high rates of recommending vaccination and high vaccination rates of their patients; (2) HCWs with high rates of recommending vaccination but low vaccination rates of their patients; and (3) HCWs with low rates of recommending vaccination and low vaccination rates of their patients. A convenience sample comprised of HCWs from each of the three categories was selected for participation in the focus group interview. After obtaining informed consent, each participant provided his/her basic demographic information, and reasons for receiving or not receiving influenza vaccination as well as reasons for recommending or not recommending influenza vaccination for their patients. The interviews were audio recorded and transcribed with the consent of focus group participants. The transcribed content was processed with NVivo 1.6.1 software. The contents were analyzed using a general inductive approach. Through a detailed reading of the raw data, the text in the transcription was coded as nodes. The nodes were first prepared through open coding based on the connections between sentences, contexts, and the meanings of the conversations. Second, the nodes were integrated and classified, and then, tree nodes were prepared around the main axis of meanings through selection. The primary subnodes were classified by combining the parent nodes and secondary subnodes. This process was repeated until the saturation of the theme was reached. Following the analysis, quotes were selected according to the representativeness of the findings and were translated from Chinese into English.

We used the Health Belief Model (HBM) [11] to describe qualitative and quantitative data in five key dimensions to assess HCWs' perceived susceptibility to influenza infection, severity of influenza illness, benefits and barriers associated with influenza vaccination, and cues to action to identify key predictors for influenza vaccination and recommendation of vaccination for their patients separately.

## 3 Results

For the quantitative survey, we enrolled 918 HCWs after informed consent and excluded 22 participants because five HCWs refused to participate in the follow‐up survey and 17 HCWs reported incomplete information on NCD status, vaccination status, or vaccination recommendation behavior. The response rate of the baseline survey was 86.2% (896/1039), and 896 were included in the final data analysis. Among the 896 surveyed primary HCWs, 64% were female, and 66% were over 35 years of age. The majority of HCWs (40%) had junior college level education, and 14.2% had lower than high school; 51.4% were nurses, and 32% had been working for more than 20 years as primary HCWs. More than half of the specialist doctors and public health doctors had junior college (*n* = 70) and above (*n* = 56) education (126/222, 56.7%), 26.6% (59/222) had high school/technical secondary school education, and 16.7% (37/222) had lower than high school education.

For the focus group interview, among the 12 HCWs who participated, seven (58.3%) were female. HCWs (75%) were aged 25–49 years, and 25% were aged ≥ 50 years. More than half of HCWs (58.3%) were clinical doctors, and others included three (25%) public health doctors and two (16.7%) public health nurses.

### 3.1 Influenza Vaccination of HCWs

Perceived susceptibility: HCWs reported high perceived risk of acquiring and transmitting influenza to themselves (89.5%), their family members (92.8%), and their patients (92.5%) due to their occupational exposure in health facilities (Table 1).

**TABLE 1:** Healthcare workers (HCWs) perspective on vaccine, occupational risk, and intervention (*N* = 896) in Xiangyang and Chibi Cities, China, 2018.

Statement	Total (*N* = 896)		Unvaccinated HCWs (*N* = 774)		Vaccinated HCWs (*N* = 122)	
	*n*	%	*n*	%	*n*	%
Attitude about vaccines						
I believe vaccines will protect me from the diseases—Yes	843	94.1	724	93.54	119	97.54
I believe vaccines are important for reducing or eliminating major communicable diseases in our country—Yes	826	92.2	711	91.86	115	94.26
I believe that acquiring immunity by contracting disease is better than getting vaccinated—Yes	561	62.6	488	63.05	73	59.84
I believe vaccinations do better than harm—Yes	808	90.2	696	89.92	112	91.80
Attitude about HCW's occupational risk in transmission of seasonal influenza						
I believe HCWs are more likely to contract influenza due to their occupational exposure in health facilities—Yes	802	89.5	692	89.41	110	90.16
I believe HCWs are more likely to transmit influenza to patients at workplace they work—Yes	829	92.5	715	92.38	114	93.44
I believe HCWs are more likely to transmit influenza to their family members—Yes	831	92.8	718	92.76	113	92.62
Intervention for influenza vaccination of HCWs						
My employers recommended or required HCWs' influenza vaccination—Yes	473	52.8	368	47.55*	105	86.07*
You were provided no‐cost influenza vaccination at workplace—Yes	241	26.9	156	20.16*	85	69.67*
You were provided on‐site influenza vaccination in workplace—Yes	610	68.1	500	64.60*	110	90.16*
My employers provided health education on influenza vaccination every year—Yes	721	80.5	607	78.42*	114	93.44*

* *p* < 0.05 for the comparisons between unvaccinated and vaccinated HCWs.

Perceived severity: In the focus group, HCWs reported “many people cannot tell the difference between the common cold and flu and believe that flu is a mild disease and therefore they do not need to waste money being vaccinated every year” (Table 2).

**TABLE 2:** Qualitative analysis of motivators and barriers for HCWs vaccination against influenza and recommendation for their patients in Xiangyang and Chibi Cities, China, 2019.

Parent nodes	Primary subnodes			
	Motivators		Barriers	
	Node	Quote example	Node	Quote example
Biomedical factors related to influenza infection and vaccine	1 Knowledge about influenza	HCWs will recommend only when they are knowledgeable about influenza and convince themselves. I believe that influenza is more serious than “Common cold.”	2 Unclear about the differences between common cold and influenza	I do not know much about influenza and have no confidence to convince my patients to get vaccinated. Many people cannot tell the difference between common cold and flu. They feel flu is a mild disease and do not need to waste money to get vaccinated every year. Healthy adults are at low risk of infection, and it will be not serious if one gets infected. Influenza more likely to attack people who are not healthy and person with chronic diseases, or children.
	3 Knowledge about influenza vaccine	Rapid and transparent information sharing of investigation results of vaccine scandal and adverse events is effective to build public confidence on vaccination. At least, influenza vaccine will relieve the symptoms of infection.	4 Effectiveness, safety, and frequency of vaccination	Compared to the EPI vaccines, I have limited knowledge about influenza vaccine. I do not know the difference between trivalent and quadrivalent vaccine and their efficiency. I am confused about the cost‐effectiveness and the necessity of getting influenza vaccination every year. Lot of HCWs are confused about the effectiveness of influenza vaccine. I do not want to recommend because it is time consuming to provide health education on influenza vaccination to patients, especially for those who has developed respiratory symptoms after receiving influenza vaccinated, and those developed side reactions after vaccination.
Social factors	5 Trust relationship between HCWs and their patients	I think patients still trust HCWs. In fact, patients care about their health and want to improve their life quality, especially for the elderly. I usually recommend influenza vaccine if patients are my relatives, friends, neighbors, and those who are familiar with me.		I do not recommend the patients who have refused my suggestion previously.
	6 Vaccination cost	I would like to recommend those with better financial status who is willing to pay out‐of‐pocket for no EPI vaccine as influenza.		I do not recommend the patients who have limited income, and they are less likely to pay for influenza vaccine.
	7 Vaccination service accessibility	I think the most effective method is to bring the vaccine and provide vaccination door‐to‐door service, and patients will accept recommendation.		I will not recommend if the vaccine clinic is bit far away from our hospital. It is inconvenient and some people even do not know where it is, especially for older adults.
	8 Vaccine related information accessibility	It is better to give patients some education brochures. During the peak season of influenza, such as December, medium report news on influenza outbreaks and infection rates. This was the opportunity to increase public awareness and influenza vaccine was quite acceptable by many people.		The health education and promotion efforts related to flu vaccine are limited.
	9 HCWs related occupational requirement and incentive mechanism	I will be more likely to recommend if my employer require HCWs providing recommendation for influenza vaccine. HCWs have duty and responsibility to prevent influenza. If there are corresponding incentive mechanisms, I would be more willing to recommend vaccines.		
	10 Social network influence	Last year, I got influenza vaccinated, and I developed only one cold. Now all my family members have been vaccinated and believe that influenza vaccine is very good to protect ourselves. If the patients or their family have experienced influenza infection or received vaccine previously, they are more likely to accept recommendation.		Some people get vaccinated, and some do not. Some people think there is not much difference between those vaccinated and those not. People may be influenced by the information in media especially internet to young people.
Subjective factors	11 Personal risk and benefit assessment	I will recommend if the patients are high risk groups for influenza complication, such as the elderly, children and NCD patients. Being a public health worker, I always recommend vaccine to people who often having colds or weak. If you tell them, they are willing to listen.		I do not want to recommend vaccine to those who look young. I normally do not recommend those NCD patients who look healthy. It's hard to know if I should recommend influenza vaccine for patients with acute infection and other chronic diseases.
	12 Vaccine efficacy knowledge	I attend a training last year related to flu and flu vaccine, which give me more confidence when recommending influenza vaccine to others.	13 Concerns related to vaccine safety	Considering vaccine scandal last year, everyone concerns about the safety of flu vaccine if I recommend it. As a public health doctor, I occasionally recommend flu vaccine, but because I did not know much about flu vaccine, and I cannot explain it clearly.

Perceived benefits: Over 90% of HCWs believe vaccines were effective and do more good than harm (Table 1). In the focus group interview, HCWs reported “influenza vaccination can at least lessen the symptoms of illness” (Table 2).

Perceived barriers: About two third (62.6%) of HCWs believed acquiring immunity by contracting influenza was better than from vaccination (Table 1). In the focus group interview, HCWs reported “compared to (Expanded Program on Immunization) EPI vaccines, I have limited knowledge about influenza vaccine,” and “I am confused about the cost‐effectiveness and the necessity for getting influenza vaccine every year” (Table 2).

Cue to action: Most HCWs (80.5%) reported their employers provided education on influenza vaccination, 68.1% had access to on‐site influenza vaccination services, 52.8% reported their employers recommended HCWs to receive influenza vaccination, and 26.9% were provided vaccination on‐site and at no cost (Table 1). The percentage of HCWs reporting the provision of these interventions for influenza vaccination at the workplace was higher among vaccinated HCWs than those who were unvaccinated.

### 3.2 HCW Recommendation for Influenza Vaccination

Perceived susceptibility: In the focus group, HCWs reported “influenza is more likely to attack people who are not healthy, persons with chronic diseases, and children”; “as a public health worker, I always recommend vaccination to people who have frequent respiratory illness or are weak; if you tell them, they are willing to listen”; and “I normally do not recommend vaccination for people who look young and healthy” (Table 2).

Perceived severity: Participants from the focus group reported “I believe that influenza is more serious than the common cold.” “I recommend vaccination when patients are at high risk for complications from influenza, such as older adults, persons with chronic disease and school‐aged children” (Table 2).

Perceived benefits: In the focus group, HCWs reported “last year, I got vaccinated for influenza, and I developed only one cold. Now all my family members have been vaccinated and believe that the influenza vaccine will protect us” and “if the patients or their family members have experienced influenza infection or previously received vaccination, they are more likely to accept influenza vaccine” (Table 2).

Perceived barriers: In the focus group, HCWs reported “I don't know the difference between trivalent and quadrivalent vaccine and their efficacy”; “I am confused about the cost‐effectiveness and the need to get influenza vaccination every year”; “many HCWs are confused about the effectiveness of the influenza vaccine”; and “as a public health doctor, I occasionally recommend flu vaccine, because I don't know much about flu vaccine, and I cannot explain it clearly” (Table 2).

Cue to action: In the focus group, HCWs reported factors related to vaccine access: “I would like to recommend influenza vaccine to those who are able to pay out‐of‐pocket for the vaccine,” and “I will not recommend vaccination when the vaccine clinic is too far away from the village clinic, because it is inconvenient to be vaccinated, especially for the elderly.” “I would be more likely to recommend vaccination if my employer required that I do so,” and “HCWs have a duty and responsibility to prevent influenza” (Table 2).

## 4 Discussion

Quantitative and qualitative data showed that primary HCWs acquired basic knowledge about influenza infection and vaccination. However, being a professional group, HCWs perceptions about influenza susceptibility and severity, and influenza vaccine side effects are limited, which may influence their behavior for vaccination and recommendation. In addition, in this study, one in seven primary HCWs reported an education lower than high school education, which may also contribute to the limited conceptions in the study group. However, HCWs understood their duty and responsibility for influenza prevention and would like to comply with occupational requirements, if any. The findings could be used to tailor health education materials by focusing on HCWs' knowledge gap and supporting the workplace requirements for HCWs vaccination and recommendation for their patients.

### 4.1 Perception of Susceptibility and Severity of Influenza Infection

Though most primary HCWs agreed that they were more likely to acquire and transmit influenza to their patients and family due to occupational exposure, two thirds of unvaccinated primary HCWs believed that they were at low risk of infection [10]. Influenza “infection” was intentionally used in the survey, as this includes both symptomatic and asymptomatic infection. One study reported that an incidence of RT‐PCR‐confirmed influenza illness among unvaccinated HCP was 9.1 per 100 person‐seasons, but the incidence more than doubled when combined with serological evidence of infection [1]. So HCWs may have underestimated their risk for influenza infection if they misinterpreted infection as illness. This suggests that health education material should use the more accurate term of “infection” not “illness” and include evidence of disease burden for HCWs. For influenza severity, the study found HCWs usually perceived seasonal influenza was not a serious disease. It is true that influenza is frequently a mild illness in healthy working‐aged adults, such as most HCWs. However, it can cause severe disease among persons with preexisting medical conditions, young children, and pregnant women. HCWs need to know that influenza is a public health problem [12]. This reminds us that education materials should provide HCWs with a full range of evidence to facilitate their understanding of influenza severity. In addition, our previous study also found that the top three reasons for HCWs getting vaccinated in descending order were protecting themselves, their patients, and their family [10]. Previous studies reported that HCWs indicate more motivation to receive influenza vaccination from self‐protection than from protection for their patients [13]. However, HCWs also acknowledged that they had an occupational responsibility for patients' safety. We believe that it is equally important for HCWs to receive vaccination for protection for their patients and suggest an approach of highlighting influenza severity of patients and patient safety in health education in the future.

### 4.2 Perception of Influenza Vaccine Benefit and Barrier

Though most HCWs believed vaccinations provide more benefit than harm, over half of the unvaccinated HCWs believed influenza infection might produce better immunity than vaccination [10]. However, natural infection comes at a cost to reduce work productivity [14, 15] and could result in further transmission to vulnerable populations at risk for severe influenza illness and death. In addition, our previous study reported that HCWs were concerned about the side effects of influenza vaccination [10]. Side effects of influenza vaccine are relatively benign [16]. Future inquiries about HCW knowledge of side effects could help inform how to approach these concerns.

### 4.3 Cues to Action

Vaccination is a complex behavior and must be understood from multiple factors, such as attitudes, beliefs, self‐efficacy, motivation, perceived threat, and social network that influence people's behavior [17], and those factors should be addressed in designing interventions. Previous studies have documented the benefits of education, offering the vaccine at no cost, providing vaccination on‐site, and requiring vaccination at the workplace in increasing coverage among HCWs [10, 18, 19, 20, 21]. Our study reports the same cues to action. First, 80.5% of primary HCWs reported that their employers provided health education on influenza vaccination every year; however, HCWs perceptions about influenza susceptibility, severity, influenza vaccine benefit, and side effects are limited. HCWs are a specific group to be targeted for health education. Compared with the public and other high‐risk groups, HCWs need to have more comprehensive information and data to support their analysis of risk and benefit for influenza vaccination. In addition, our previous study found one third of HCWs did not know the national policy for influenza vaccination [10]. This suggests health education materials could include national and international policies and guidelines on influenza vaccination in the future. In addition, some senior traditional Chinese doctors who completed limited medical training after graduating from high school and below are permitted to serve as primary HCWs in the rural and remote areas in China. It highlighted the significance of strengthening health education on influenza and vaccination for this group of HCWs in China. Second, HCWs have a professional or moral obligation to protect their patient's safety and minimize harm [22, 23]. Although mandatory workplace requirements for influenza vaccination for HCWs are the most effective in increasing influenza vaccination [10, 18, 21], it presents both ethical and logistical challenges for implementation. China has no national mandatory policy for workplace vaccination of HCWs. Our previous study found HCWs frequently reported no employer requirements as a reason for not recommending the vaccine to patients [10]. HCWs reported their willingness to follow occupational requirements. Implementing workplace requirements in addition to providing free, on‐site influenza vaccination and education would facilitate acceptance of and recommendation for influenza vaccination among HCWs and their peers. However, HCWs' autonomy should be considered when workplace requirements for vaccination are implemented among HCWs.

### 4.4 Limitations

This study is subject to several limitations. First, enrolled primary HCWs were from two cities in central China and may not be representative and generalizable to China. Second, due to low vaccination coverage, we did not include vaccination status as one criterion to categorize the clinicians for the focus group interview. Third, we collected the quantitative data from a baseline survey, and the qualitative data were collected from a focus group interview after an intervention of training and workplace requirement for influenza vaccination recommendation for their patients. We understand inconsistent timing between the quantitative and qualitative data and chose this strategy for the following reasons: (1) The study aim was to identify unmet needs of primary HCWs over time, not to compare quantitative and qualitative data; (2) the quantitative data from the baseline survey may be more generalizable and less contaminated by the study interventions; and (3) the qualitative data were used to verify quantitative findings over time, and we found the same misconceptions continued to exist after the intervention. Lastly, the study was conducted before the COVID‐19 pandemic, so HCWs' acceptance and practices of vaccine may have been altered by their experience in the pandemic. A postpandemic follow‐up study of these HCWs would be possible to explore the current status.

## 5 Conclusions

Our study identified several predictors that could guide future interventions to improve influenza vaccination uptake and recommendation among primary HCWs by enhancing health education materials to include comprehensive information on the burden of influenza disease, side effects of vaccination, and national and international policies and guidelines on influenza vaccination, introducing workplace requirements for influenza vaccination and recommendation among HCWs in addition to providing cost‐free, on‐site vaccination.

## Author Contributions

**Jing Fan:** methodology, validation, formal analysis, investigation, data curation, writing – original draft, writing – review and editing, project administration. **Ran Zhang:** methodology, validation, investigation, writing – original draft, writing – review and editing. **Qianyue Zhang:** methodology, validation, formal analysis, writing – original draft, writing – review and editing. **Shu Cong:** methodology, investigation, writing – review and editing. **Ying Song:** conceptualization, methodology, investigation, writing – review and editing, project administration, funding acquisition. **W.William Schluter:** methodology, writing – review and editing. **Suizan Zhou:** methodology, investigation, writing – review and editing. **Xuping Song:** methodology, writing – review and editing. **Wenjing Wang:** methodology, writing – review and editing. **Liwen Fang:** conceptualization, methodology, investigation, writing – review and editing, supervision, project administration, funding acquisition.

## Ethics Statement

The study was conducted in accordance with the Declaration of Helsinki and approved by the Ethics Review Committee of the National Center for Chronic and Non‐Communicable Disease Control and Prevention, China CDC (Project No. 201820; date approved: April 27, 2018), and the Institutional Review Board of the USCDC (Human Subjects Research‐Not Engaged).

## Consent

Written informed consents were obtained from all primary HCWs in the study.

## Conflicts of Interest

The authors declare no conflicts of interest.

## Data Availability

The datasets presented in this article are not readily available because they were used under license for the current study. Requests to access the datasets should be directed to National Center for Chronic and Non‐Communicable Disease Control and Prevention, Chinese Center for Disease Control and Prevention (China CDC), Xicheng District, 100050, Beijing, China.
